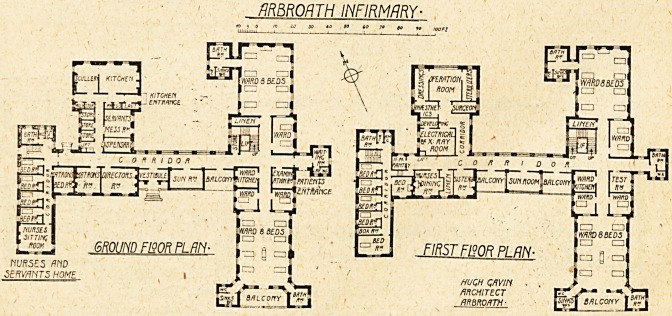# The New Arbroath Infirmary

**Published:** 1916-11-04

**Authors:** 


### A GROUP OF PLANS.

IN the following pages we illustrate and describe plans of various new buildings and reconstructions, including a new general infirmary; a reconstructed children's hospital; a new large open-air sanatorium, the design for which presents features of special interest; a small cottage hospital, built in connection with a manufacturing colony, and managed by a committee nominated half by the company and half by the staffs of office and works; and an important American hospital of 225 beds.

### THE NEW ARBROATH INFIRMARY.

It would appear that the original intention of the Infirmary directors was to reconstruct the old buildings, but as subscriptions received for this purpose exceeded the sum required, the decision was arrived at to

BLOCK PLAN·

pull down the old building and erect an entirely new one. The old building was of an obsolete type, and could not have been successfully converted so as to conform to modern requirements. The decision taken by the directors was therefore undoubtedly a wise one.

The new infirmary may be described as consisting of three blocks: the ward block, the administration and operation block, and the nurses' home, all three being connected up by a corridor.

With the exception of an attic storey over the nurses' home the whole building is two storeys in height.

The ward block contains on each floor two wards of eight beds each, a ward for two beds, and two single-bed wards. Medical cases occupy the ground floor and surgical cases the upper floor, the male patients occupying one end of the block, female cases the other. One ward kitchen, testing-room, and linen-room serves both wards. On the ground floor is the entrance for patients, with waiting-room and examination-room.

The south wards are provided with a balcony, and the sanitary offices are arranged in two wings at the angles of the ward. The sanitary offices for the north wards are placed in one wing at the north-west side. A bathroom and w.c. are provided in the centre of the east side for the use of the small wards.

On the south side of the corridor connecting the ward block with the administration and theatre block are two spacious balconies and a sun-room.

The administration on the ground floor provides on the south side a directors' room and the matron's sitting-room. At the back is the dispensary, store-rooms, and kitchen offices.

On the upper floor in front are sister's room, linen store, and nurses' dining-room. At the back is the operation unit. This comprises the theatre with dressings-room and sterilising-room on either side, anæsthetic-room, surgeon's room, and electrical and *x*-ray department, with dark-room for developing. There is no communication between the anæsthetic-room and the theatre except by passing through the dressings-room; an objectionable and much too common fault in theatre design.

The east wing is entirely devoted to nurses and servants, and provides a very compact home for the purpose.

The hospital was designed by Mr. Hugh Gavin, of Arbroath, with the expert advice of Dr. Mackintosh, C.V.O.

## Figures and Tables

**Figure f1:**
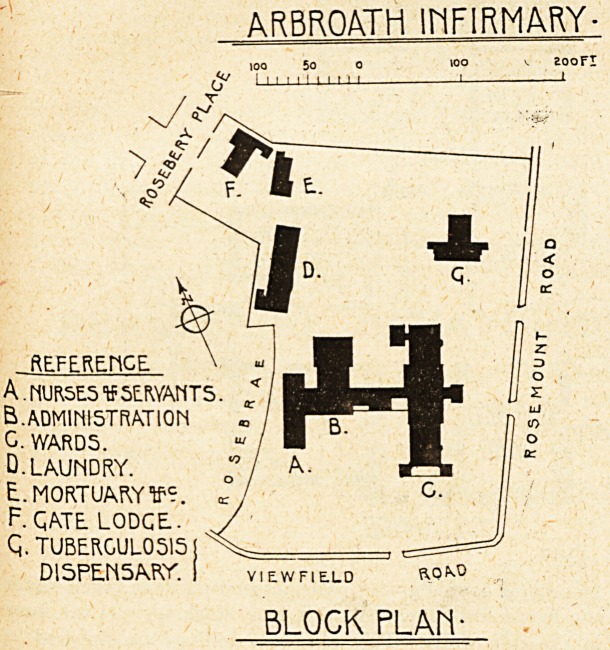


**Figure f2:**